# Congenital unilateral athelia associated with mammary Paget’s disease, high-grade ductal carcinoma *in situ* and microinvasive breast cancer: a case report

**DOI:** 10.3389/fonc.2026.1829248

**Published:** 2026-08-20

**Authors:** Yufei Xiao, Wei Chen, Ruoling Han, Yaxin Chu, Zerou Guo

**Affiliations:** The Department of Ultrasound, Fourth Hospital of Hebei Medical University, Shijiazhuang, China

**Keywords:** athelia, HER-2, high-grade ductal carcinoma *in situ*, immunohistochemical findings, mammary Paget’s disease, mammographic findings, ultrasonography

## Abstract

**Background:**

Congenital unilateral athelia is an extremely rare breast malformation, most commonly associated with ectodermal dysplasia or systemic congenital syndromes. Isolated congenital athelia without concurrent malformations is even rarer, and its long-term neoplastic risk remains poorly characterized.

**Case description:**

We report a 57-year-old postmenopausal woman with congenital isolated right athelia who presented with a 2-year history of bilateral breast masses. Multimodal imaging revealed multifocal lesions in the right breast, and postoperative pathology confirmed synchronous mammary Paget’s disease, extensive high-grade comedo-type ductal carcinoma *in situ* (DCIS), and microinvasive breast carcinoma. The patient underwent right mastectomy with sentinel lymph node biopsy followed by adjuvant chemotherapy, with no evidence of recurrence during 12-month follow-up.

**Key findings:**

This is the first reported case of isolated congenital athelia complicated with triple synchronous epithelial breast malignancies. Congenital nipple absence introduces substantial challenges to preoperative imaging localization, intraoperative margin assessment, and postoperative surveillance. Ultrasonography, with flexible scanning planes and real-time performance, shows unique advantages in disease management for such conditions.

**Conclusion:**

Although no causal relationship is established between congenital athelia and breast carcinogenesis, careful clinical and imaging evaluation is mandatory for patients with congenital nipple-areolar abnormalities. Rational application of multimodal imaging is critical for early diagnosis and long-term surveillance in this rare population.

## Introduction

The absence of the nipple, termed athelia, is a rare clinical condition that early investigators generally attributed to ectodermal dysplasia during embryonic development. In 1993, Triolo et al. explicitly reported cases of anhidrotic ectodermal dysplasia associated with nipple agenesis (1). Subsequent clinical observations have linked nipple-areolar complex (NAC) agenesis to a spectrum of congenital syndromes, including Raas–Rothschild syndrome (facial clefting and skeletal anomalies) (2), scalp–ear–nipple syndrome (cutaneous and auricular dysplasia with hypoplastic nipples) (3), and Poland’s syndrome (pectoral muscle aplasia and syndactyly) (3). These syndromic forms arise from either localized embryonic ischemic injury or germline mutations, which disrupt mesenchymal induction or vascular supply to the ectodermal mammary placodes along the mammary ridge, arresting nipple bud proliferation, invagination, and vascularization. In contrast, isolated congenital athelia—defined as complete NAC agenesis without other systemic or musculoskeletal malformations—is exceedingly rare, with only 5 cases reported in the literature to date (4–8). These cases show a unilateral-to-bilateral ratio of approximately 2:3, predominantly affecting adolescent females aged 16–24 years. Pathogenetically, isolated athelia arises from localized developmental arrest of the nipple anlage, with normal development of the mammary gland parenchyma, ductal system, chest wall, and systemic ectodermal structures. No familial clustering or pathogenic mutations in known mammary developmental genes have been identified in this population.

Prior research on congenital athelia has largely focused on embryological mechanisms and reconstructive surgical techniques, while data on long-term breast neoplastic risk remain scarce. Herein, we present the first case of isolated congenital unilateral athelia associated with synchronous mammary Paget’s disease, high-grade DCIS, and microinvasive carcinoma, and systematically discuss the clinical challenges and imaging management strategies for this rare condition.

## Case report

**Figure 1:**
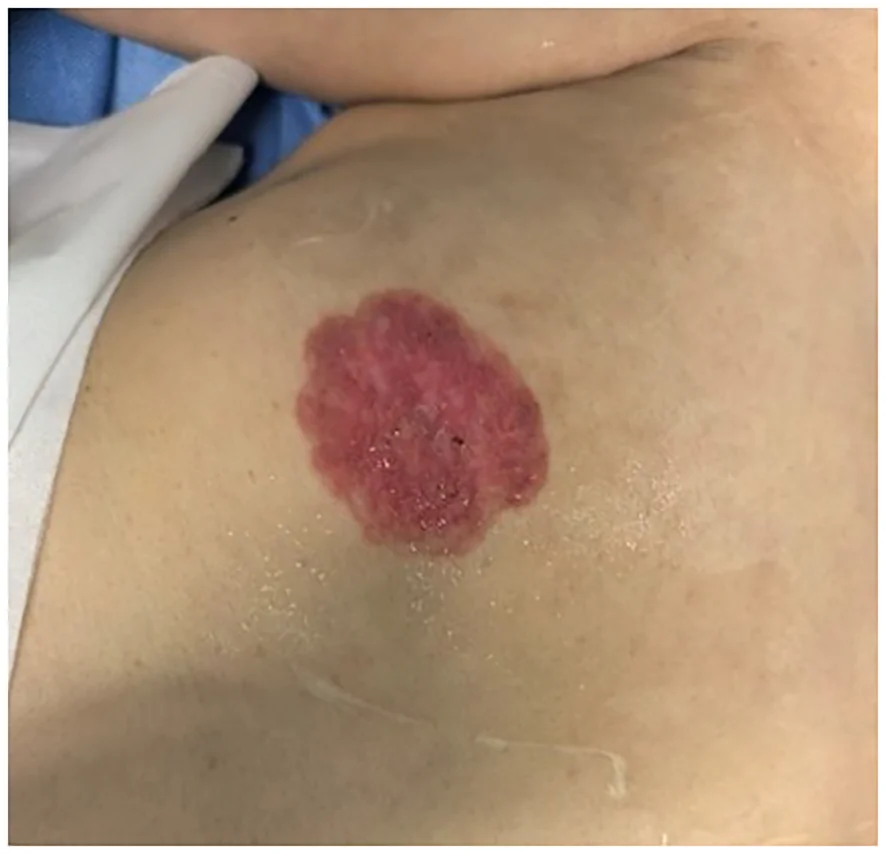
No nipple was present on the right breast. Erythema with skin ulceration was observed in the central area. Neither a dimple sign nor peau d’orange changes were noted.

Clinical Presentation: A 57-year-old woman complained of discovering bilateral breast masses for 2 years. Her menarche occurred at 12 years of age, and she reached menopause at 50 years, with a regular 30-day menstrual cycle. She had one full-term delivery and one spontaneous abortion, with no family history of breast or ovarian cancer. Physical examination revealed symmetric bilateral breasts with complete congenital absence of the right NAC. Focal skin erythema, swelling, and superficial ulceration were noted in the central right breast, without superficial varicosities, dimple sign, or peau d’orange changes (**Figure 1**). No distinct masses were palpable in either breast. The thoracic cage was symmetric and undeformed, with no abnormalities of the skull, ears, nasal cavity. No lymph nodes were palpable in the bilateral axillae and bilateral supraclavicular regions.

**Figure 2:**
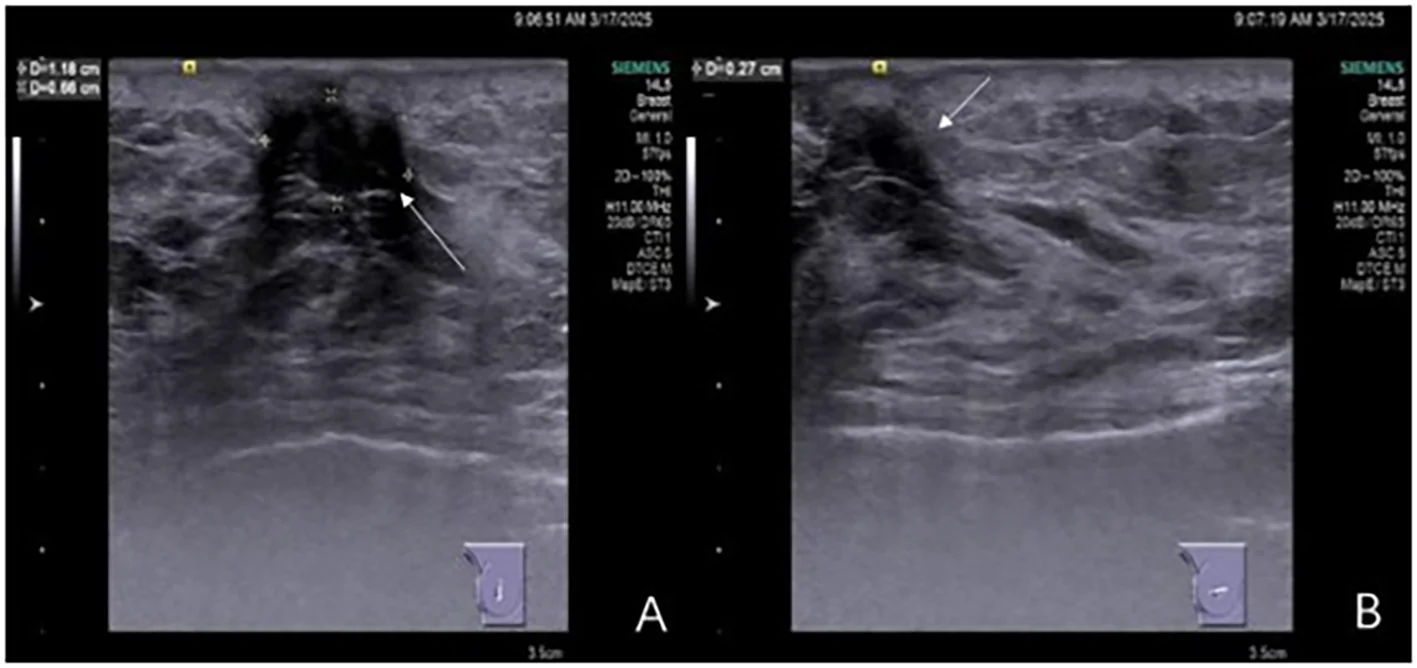
Ultrasonographic images obtained with a Siemens ACUSON S2000 system equipped with a 14L5 linear array transducer. **(A)** A hypoechoic nodule (arrow) and several dilated ductal echoes were observed in the subcutaneous layer. **(B)** A hypoechoic nodule (arrow) was detected in the upper outer glandular layer of the right breast, with an ill-defined margin and focal angular features. Duct–like structures were noted around the lesion.

Imaging Findings: Breast ultrasonography demonstrated skin thickening in the right breast. A subcutaneous hypoechoic lesion measuring approximately 1.3 × 1.2 × 0.7 cm was identified (**Figure 2A**), characterized by irregular margins and indistinct borders. Several dilated ducts were noted within and beneath the lesion, extending toward the upper inner quadrant. The largest duct measured approximately 0.27 cm in diameter and showed reduced acoustic transmission with patchy hyperechoic foci within the lumen. Color Doppler Flow Imaging (CDFI) demonstrated moderately increased vascularity within and around the lesion. Additionally, a hypoechoic nodule was identified in the upper outer quadrant of the right breast, measuring approximately 1.1 × 0.9 × 0.5 cm (**Figure 2B**). The nodule appeared roughly regular in shape, with partially indistinct margins and focal angulation. Peripheral duct-like structures were visible, and CDFI demonstrated distinct internal vascularity.

**Figure 3:**
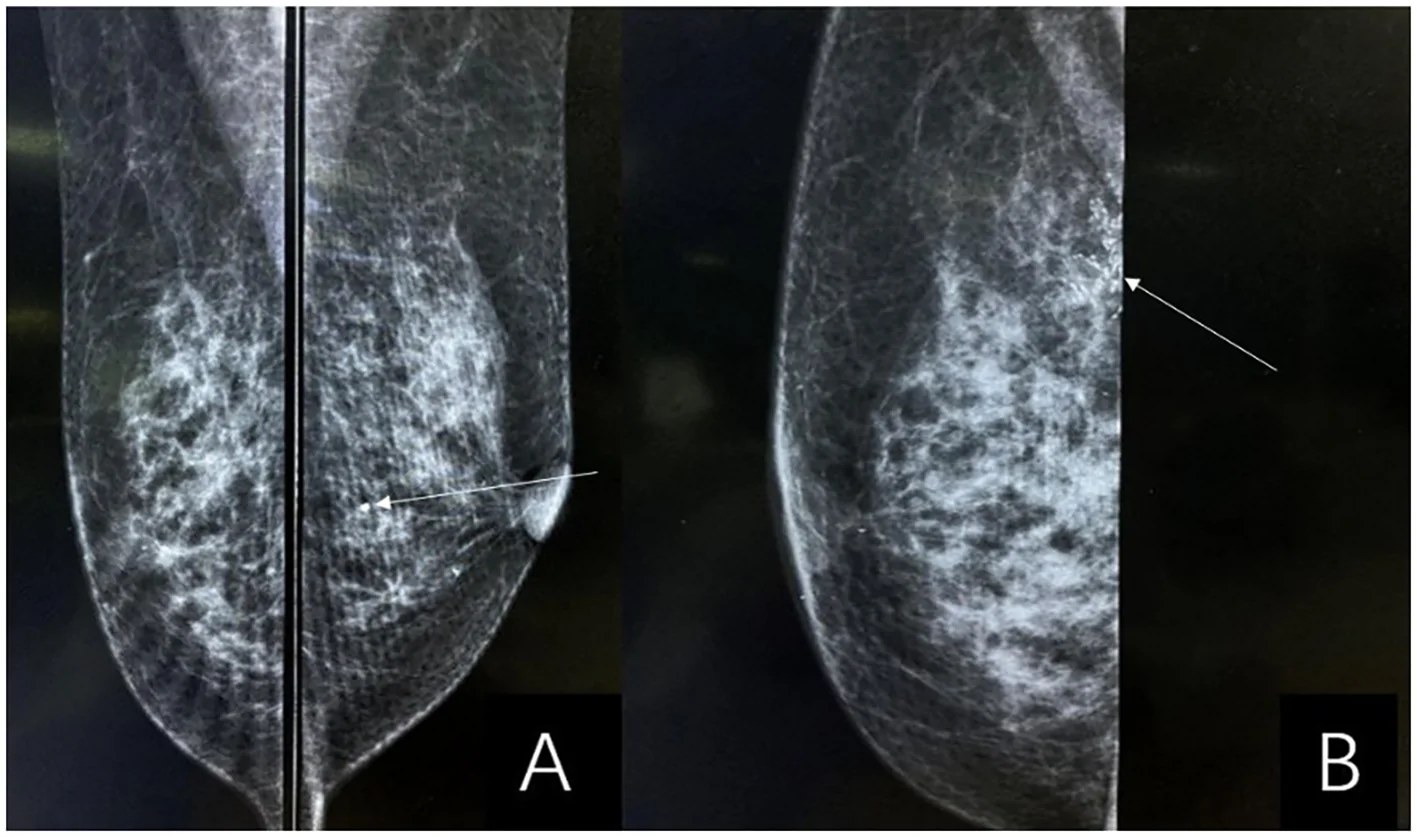
Mammography acquired with GE Senographe Pristina Hygeia system. **(A)** The right nipple was not visualized. Skin thickening was noted in the right areolar region, with indistinct subcutaneous fat planes. Multiple fine pleomorphic microcalcifications (arrow) were observed in the areolar, lower, and outer quadrants of the right breast. **(B)** On the mediolateral (ML) view, clustered coarse heterogeneous calcifications(arrow) were seen in the upper quadrant of the right breast.

Mammographic examination revealed thickening of the skin over the right areolar region and absence of the right nipple, with obscuration of the subcutaneous fat layer. Multiple fine, pleomorphic microcalcifications were observed in the right areolar region and in the lower-inner and outer quadrants, with predominant clustering in the latter areas. Additionally, the mediolateral (ML) view demonstrated a cluster of irregular calcifications in the upper quadrant of the right breast (**Figure 3**).

**Figure 4:**
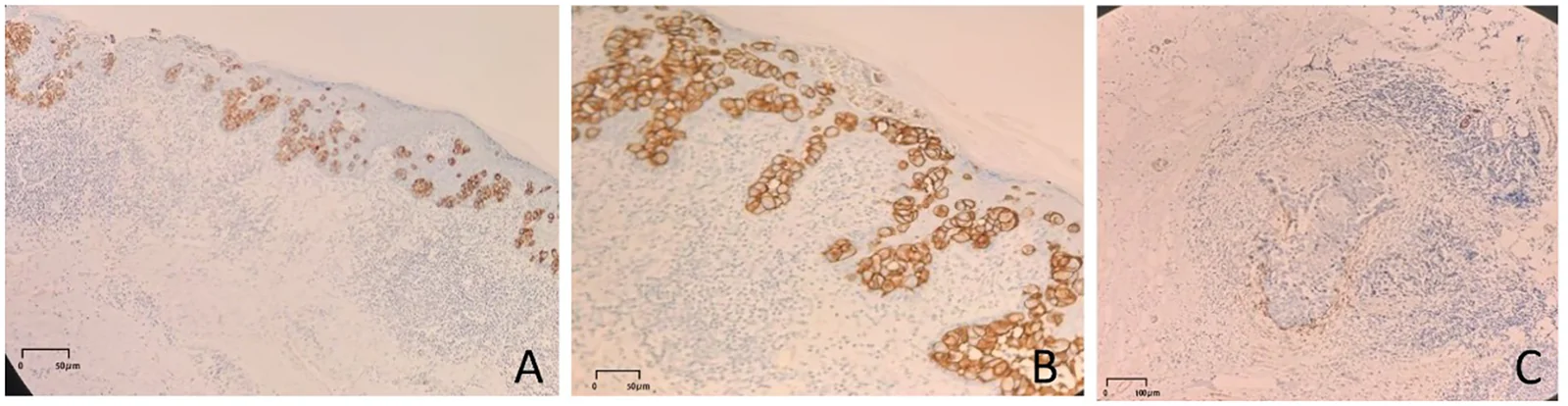
Immunohistochemical staining of the right breast mass and partial areolar tissue. **(A)** CK7(+); **(B)** HER2(+); **(C)** Calponin staining: focal loss of myoepithelial cells, indicative of microinvasion.

Pathological Findings: The patient initially underwent local excision of right breast lesions under local anesthesia on March 17, 2025. Postoperative paraffin pathology with immunohistochemistry (IHC) showed (**Figure 4**):

1. Right areolar skin lesion: Mammary Paget’s disease with underlying high-grade DCIS; IHC profile: estrogen receptor (ER) (−), progesterone receptor (PR) (−), cytokeratin 7 (CK7) (+), E-cadherin (+), cytokeratin 20 (CK20) (−), cytokeratin 5/6 (CK5/6) (−), Ki-67 (60% positive), S100 (−), p63 (−), human epidermal growth factor receptor 2 (HER2) (3+).
2. Medial right breast lesion: High-grade comedo-type DCIS; IHC profile: ER (−), PR (−), HER2 (3+, intraductal), Ki-67 (30% positive), E-cadherin (+), CK5/6 (intact), p63 (intact).
3. Lateral right breast lesion: Microinvasive breast carcinoma (maximum invasive focus diameter < 1 mm) with extensive surrounding high-grade comedo-type DCIS; IHC profile: ER (−), PR (−), HER2 (3+, both intraductal and invasive foci), Ki-67 (40% positive), E-cadherin (+), CK5/6 (focal loss), p63 (focal loss), calponin (focal loss).

Treatment and Follow-up: On March 25, 2025, the patient underwent a right mastectomy with sentinel lymph node biopsy under general anesthesia. The excised specimen measured 17 cm × 16 cm × 3 cm, including a 14 × 6 cm elliptical skin flap. Pathological evaluation confirmed focal residual high-grade DCIS at the first primary incision site, focal atypical ductal hyperplasia at the second incision site, and no residual tumor at the third incision site. All four sentinel lymph nodes were negative for metastasis. The final pathological stage was pT1miN0. After multidisciplinary team consultation, adjuvant chemotherapy was administered given the extensive lesion scope, high histological grade, and patient preference. The modified regimen consisted of nab-paclitaxel 310 mg plus cyclophosphamide 1.0 g intravenously every 3 weeks, with concurrent proton pump inhibitor and antiemetic prophylaxis.

The patient was followed up every 3 months with a planned 5-year surveillance period. At the latest follow-up on March 25, 2026, the surgical wound had healed well with no local recurrence. Breast ultrasonography showed no space-occupying lesions in the right chest wall, and chest computed tomography (CT) revealed postoperative changes with multiple benign-appearing pulmonary micronodules in the right lung. The patient remained in good general condition with no evidence of distant metastasis.

## Discussion

This report presents an extremely rare case of congenital unilateral isolated athelia complicated by synchronous mammary Paget’s disease, extensive high-grade comedo-type DCIS, and microinvasive breast carcinoma in a postmenopausal woman. To our knowledge, this is the first documented case of triple synchronous epithelial breast malignancies arising in the setting of isolated congenital athelia. The following discussion contextualizes the findings against existing literature, delineates the clinical challenges associated with nipple agenesis in breast cancer care, explores underlying pathogenic mechanisms, and outlines diagnostic and management considerations for this rare patient population.

The co-occurrence of congenital nipple agenesis and breast malignancy poses unique clinical challenges across the entire care continuum from preoperative diagnosis to long-term surveillance. Preoperatively, as the NAC serves as the primary anatomical landmark for central breast lesion localization, its absence compromises standard mammographic performance, leading to incomplete glandular coverage and reduced sensitivity for central lesions due to congenital breast asymmetry and obscured subcutaneous fat planes (8). Meanwhile, the lack of a nipple reference point on ultrasonography impairs differentiation between physiological skin thickening and pathological infiltration as well as tracing of central ductal anatomy, elevating the risk of missed multifocal lesions. Intraoperatively, loss of the NAC as a reference for medial surgical margin definition renders margin assessment highly subjective for both breast-conserving surgery and mastectomy, increasing the risk of positive resection margins or unnecessary normal tissue resection, and particularly reducing the feasibility of achieving negative margins for central lesions via breast-conserving approaches. For postoperative reconstruction, conventional imaging cannot reliably quantify breast gland volume and skin envelope dimensions in the setting of anatomical asymmetry, which may cause overestimation of residual glandular tissue and consequent postoperative bilateral breast asymmetry. For long-term surveillance, patients with congenital athelia lack key clinical warning signs of recurrence such as nipple discharge, retraction and eczematous changes and rely entirely on imaging; the absence of standardized baseline imaging further complicates differentiation between postoperative changes and recurrent lesions, increasing misdiagnosis risk.

From a molecular perspective, the synchronous presentation of multiple lesions in this case can be explained by a shared clonal origin and oncogenic pathway activation. The three synchronous lesions in this case exhibited an identical immunophenotype (ER-negative, PR-negative, HER2 (3+), Ki-67 index ≥30% and positive E-cadherin expression), strongly supporting a monoclonal origin from mammary ductal epithelium. Postmenopausal women exhibit markedly decreased serum estrogen and progesterone levels, which attenuate hormone receptor-mediated oncogenic signaling. Amplification of the HER2 gene aberrantly activates canonical pro-oncogenic cascades including the PI3K/AKT/mTOR pathway. Meanwhile, HER2 engages in crosstalk with estrogen receptors to establish a positive feedback loop, disrupting the homeostatic balance of normal cell cycle progression, apoptosis and cellular metabolism (9), and thereby driving sustained aberrant proliferation of ductal epithelial cells. This molecular mechanism partially accounts for the extensive high-grade ductal carcinoma *in situ* observed in the index case. Furthermore, from the perspective of breast developmental embryology, the human breast arises from the embryonic ectodermal mammary ridge, and congenital nipple absence results from arrested mammary ridge development (10). This developmental arrest directly leads to congenital atresia of the external orifice of the main lactiferous duct, which in turn causes secretion stasis and the production of chronic inflammatory mediators—factors that ultimately increase the risk of abnormal ductal epithelial hyperplasia. Abnormal activation of canonical signaling pathways such as PI3K in postmenopausal women, combined with chronic inflammatory stimuli, further endows high-grade ductal carcinoma *in situ* (DCIS) with invasive features including intraductal spreading and multifocal extension. Persistent stimulation of these factors ultimately triggers the initiation of microinvasion (9).

From an epidemiological perspective, congenital athelia can be broadly categorized into syndromic and isolated subtypes, with markedly distinct breast cancer risk profiles supported by existing evidence. Syndromic athelia, as a phenotypic component of multisystem developmental disorders, is consistently associated with elevated long-term breast cancer risk. For instance, ulnar-mammary syndrome, caused by germline TBX3 mutations and characterized by nipple hypoplasia, mammary gland hypoplasia and upper limb skeletal anomalies, confers a substantially higher lifetime breast cancer risk relative to the general population. A 2020 systematic review demonstrated that up to 32% of affected females develop breast cancer by 60 years of age, with mean age at onset 8–10 years earlier than sporadic breast cancer (11). Similarly, patients with Poland’s syndrome, which frequently manifests with nipple hypoplasia or complete athelia accompanied by pectoral muscle aplasia, exhibit a 2.3-fold higher relative risk of ipsilateral breast cancer in long-term follow-up. This elevated risk is attributed to chronic ductal stasis and aberrant mammary parenchymal development secondary to embryonic ischemic injury (11). Mechanistically, the increased neoplastic risk in syndromic forms arises from the dual effects of germline mutations disrupting mammary developmental pathways, and structural ductal anomalies that trigger chronic inflammation and progressive epithelial hyperplasia. In contrast, isolated athelia has historically been considered a benign anatomical variant with no proven neoplastic risk, as all 5 previously reported cases involved young females with no evidence of breast malignancy during follow-up (4–8). However, the present case—with synchronous triple malignant lesions in a postmenopausal woman with isolated athelia—challenges this assumption. Embryologically, isolated athelia results from localized arrest of nipple anlage development, which causes congenital atresia of the main lactiferous duct orifice. This structural defect leads to secretion stasis and chronic inflammatory mediator production, which may increase the risk of ductal epithelial hyperplasia and malignant transformation over decades, particularly in the context of postmenopausal oncogenic pathway activation. While population-level risk data are lacking, this case suggests that isolated athelia may not be entirely benign, and long-term surveillance is warranted.

Against this background, the clinical significance of the present case becomes more evident when compared with previously reported cases of isolated athelia. All prior cases were diagnosed in young females aged 16 to 24 years, with clinical management centered on aesthetic reconstruction and anatomical characterization (12); none reported concurrent breast malignant lesions. The present case is the first to describe synchronous triple epithelial malignancies in the setting of isolated congenital athelia, and the first report of isolated athelia presenting with malignant manifestations in a postmenopausal patient. These findings expand the clinical spectrum of this rare disorder and underscore its previously unrecognized long-term neoplastic potential.

Mammary Paget’s disease typically manifests as an eczematous change of the skin over the nipple and is frequently associated with underlying ductal carcinoma *in situ* or invasive ductal carcinoma (13). Based on the systematic clinicopathological review by Zeng et al. (14), central erythematous and ulcerative cutaneous lesions arising in patients with congenital athelia require rigorous differentiation between benign eczematous dermatoses, primary cutaneous Bowen disease, and mammary Paget’s disease complicated by underlying DCIS. Benign nipple eczema, including atopic and irritant/allergic contact dermatitis, typically presents with bilateral pruritic plaques responsive to topical corticosteroids, showing only superficial spongiotic epidermal changes on histology without intraductal neoplastic lesions or mammary gland abnormalities on multimodal imaging. Bowen disease manifests as an isolated cutaneous lesion confined to the epidermis, characterized by full-thickness squamous atypia and an immunophenotype positive for high-molecular-weight cytokeratin CK5/6 and p63 yet negative for CK7 and HER2, with no concomitant breast ductal pathology. In contrast, mammary Paget’s disease associated with DCIS presents as unilateral, treatment-refractory central skin erosion, accompanied by mammographic or ultrasonographic evidence of dilated lactiferous ducts and clustered microcalcifications; histologically, characteristic CK7- and HER2-positive intraepidermal Paget cells can be identified, often alongside underlying intraductal carcinoma. Given the absence of nipple-based clinical landmarks and typical warning signs in athelia patients, combined breast imaging and deep biopsy covering both skin and subcutaneous glandular tissue are essential to rule out synchronous DCIS mimicking benign inflammatory skin disease.

Moreover, recent studies have demonstrated that ductoscopy combined with cytological examination of ductal lavage fluid can significantly improve the early detection rate of intraductal lesions (15). However, as an invasive procedure, ductoscopy carries certain risks of bleeding and infection and can only be performed when ductal dilatation is clearly visible upon inspection. In the present case, the patient had a congenital athelia accompanied by erythema, swelling, and superficial skin ulceration, rendering ductoscopy unsuitable. Mammography yields highly standardized imaging and enables clear visualization of mammary glands and intraductal microcalcifications. Nevertheless, for patients with congenital breast malformations, congenital nipple absence renders standard projection positions unfeasible, which may partially lead to incomplete coverage of glandular tissue during acquisition. MRI is not limited by nipple or thoracic wall deformities; however, it cannot facilitate real-time puncture biopsy and carries substantial medical costs. Under such clinical circumstances, ultrasonography demonstrates distinct advantages as a radiation-free, position-flexible, real-time and relatively accurate imaging modality without strict positioning requirements. This case report documents, from the perspective of a sonographer, the sequential imaging findings, clinical management, and postoperative follow-up of a patient with congenital nipple absence.

Notably, ultrasound offers distinct advantages in the evaluation and management of such patients: first, it allows for flexible multi-angle and multi-planar scanning of abnormal nipple regions (16). Second, contrast-enhanced ultrasound (CEUS) as a valuable complementary modality for evaluating multifocal intraductal lesions in patients with congenital athelia. CEUS enables accurate delineation of lesion extent by visualizing microvascular perfusion of intraductal neoplastic tissue, clearly demarcating the boundary between malignant epithelium and normal ductal mucosa. For athelia patients lacking nipple landmarks, CEUS can precisely map the longitudinal spread of DCIS along ductal systems, reducing the underestimation of lesion scope commonly seen with conventional ultrasound (17, 18). In addition, ultrasound examination possesses real-time capability, enabling continuous monitoring of tumor progression over a specific period and facilitating timely image-guided puncture for pathological confirmation when clinically indicated (19). Under the condition of abnormal breast anatomical structure, these advantages endow ultrasound with important value in early detection and diagnosis of breast malignant lesions and long-term postoperative prognostic follow-up.

## Limitation

As a single retrospective case report, it cannot establish a causal relationship between congenital athelia and breast carcinogenesis; the co-occurrence may represent an incidental association rather than a pathogenic linkage. The proposed mechanism of chronic ductal stasis promoting malignant transformation remains speculative, with no direct histological or molecular evidence derived from this case.

## Data Availability

The original contributions presented in the study are included in the article/supplementary material. Further inquiries can be directed to the corresponding author.
